# Characterisation of the *Mycobacterium tuberculosis* alternative sigma factor SigG: Its operon and regulon^☆^

**DOI:** 10.1016/j.tube.2013.05.005

**Published:** 2013-09

**Authors:** Alison Gaudion, Lisa Dawson, Elaine Davis, Katherine Smollett

**Affiliations:** Division of Mycobacterial Research, MRC National Institute for Medical Research, The Ridgeway, Mill Hill, London NW7 1AA, UK

**Keywords:** *Mycobacterium tuberculosis*, Sigma factors, SigG, DNA damage response

## Abstract

A major step in the pathogenesis of *Mycobacterium tuberculosis* is the ability to survive inside macrophages, where it is exposed to a number of DNA damaging agents. The alternative sigma factor SigG has been shown to be upregulated by DNA damaging agents and by macrophage infection, but not to regulate genes of the DNA repair pathway. Here we show that SigG is expressed from at least two promoters, the most dominant of these being the DNA damage inducible RecA_Ndp promoter. This promoter is located within the annotated coding region of SigG and so the correct translational start site was determined experimentally and found to be 114 bp downstream of the annotated start site. Examining the gene expression profile of a SigG over-expression strain found a small number of genes to up-regulated, two of these encoded proteins containing glyoxylase-like domains.

## 1 Introduction

Despite the availability of treatments and a vaccine *Mycobacterium tuberculosis* remains a major cause of human disease worldwide and was responsible for 1.4 million deaths in 2011.^46^ During the course of infection and transmission to new hosts *M. tuberculosis* is exposed to a number of stresses and its ability to adapt to these stresses is a key component of its survival. Not surprisingly the genome sequence of *M. tuberculosis* revealed over 100 genes encoding regulatory proteins involved in gene expression, including 13 sigma factors.^9,12^ Sigma factors are components of RNA polymerase that contain the promoter recognition domains. There are several different classes of sigma factor ranging from housekeeping sigma factors to the alternative sigma factors, which respond to specific external stimuli (reviewed in Helmann et al.^23^). The *M. tuberculosis* genome encodes 10 alternative sigma factors^38^; here we examine the alternative sigma factor, SigG.

Determining the conditions that induce expression of a particular alternative sigma factor can be useful in designating function. SigG is induced by DNA damage and is part of the RecA independent DNA damage response.^36,39^ There are two known pathways for induction of DNA repair genes in response to DNA damage in *M. tuberculosis*.^15,36^ One of these is the classical SOS response that is controlled by LexA and RecA controlling genes involved in DNA damage induced mutagenesis, cell cycle control and containing phage attachment sites.^14,17,29,41^ The other is the RecA-independent response that controls the majority of DNA damage inducible genes, including many of the genes involved in DNA repair.^36^ A DNA motif resembling a non-canonical promoter common to a number of genes regulated in this way has been identified leading to the suggestion that these genes were controlled by an alternative sigma factor.^21^ However, SigG was found not to control transcription of either the SOS or RecA-independent response genes, and mutation of *sigG* did not increase sensitivity to DNA damage.^39^ The RecA-independent DNA damage response was subsequently found to be regulated by the Clp protease regulator ClpR.^45^

We sought to determine a role for SigG both by looking at the control of its expression and by determining the genes that SigG itself regulates. In our previous work we attempted to compare gene expression in a *sigG* mutant strain to wild-type *M. tuberculosis* H37Rv but found no significant differences,^39^ possibly due to SigG being the lowest expressed of all sigma factors under normal growth conditions.^25^ Therefore, in this study we examined the regulon of *sigG* using an over-expression strain. We found that instead of controlling genes involved in DNA repair it controls genes with a potential role in detoxification.

## 2 Materials and methods

### 2.1 Bacterial strains and culture conditions

The mycobacterial strains used were *Mycobacterium smegmatis* mc^2^155,^42^ *M. tuberculosis* wild-type strain H37Rv^31^ *sigG* mutant strains in H37Rv Δ*sigG*1 and Δ*sigG*2,^39^ and a *sigG* operon deletion in H37Rv Δ*sigG*WO (this study). Mycobacterial cultures grown in Dubos medium (Difco) supplemented with albumin and 0.2% glycerol or on Difco Middlebrook 7H11 agar plates (Beckton Dickenson) supplemented with 4% albumin and 0.5% glycerol. *M. tuberculosis* liquid cultures were grown at 37 °C in a rolling incubator at 2 rpm. All procedures with live *M. tuberculosis* were carried out under ACDP containment level 3 conditions. Antibiotics were added as appropriate: kanamycin was used at 25 μg ml^−1^, hygromycin was used at 50 μg ml^−1^. To induce DNA damage cultures were divided into two aliquots at an OD_600_ 0.3–0.4 and one sample was treated with 0.02 μg ml^−1^ mitomycin C for 24 h. The other sample was incubated in parallel without treatment to provide an uninduced control. The plasmids and primers used in this study are described in Supplementary Tables 1 and 2 respectively. Site-directed mutagenesis was performed using the Quikchange site-directed mutagenesis (SDM) kit (Stratagene). All plasmids were verified by DNA sequencing.

### 2.2 Protein preparation and antibody production

Recombinant SigG was produced by expression of His-tagged *M. tuberculosis* SigG from plasmid pJH05 in *Escherichia coli* strain Tuner. Protein was purified using an ÄKTA prime (Amersham Biosciences) first using a nickel-loaded HiTrap chelating HP column (GE Healthcare), followed by purification with a HiLoad 26/60 Superdex 200 prep grade gel filtration column (GE Healthcare). Pure SigG was then used to immunise rabbits to produce polyclonal anti-SigG antibody by BioServ UK Ltd (Sheffield University); specificity was determined by Western blot against *M. tuberculosis* cell free extract.

### 2.3 Preparation of cell free extracts, Western blot and *β*-galactosidase assays

Cell free extracts were produced as described previously.^14^ The supernatants were filtered through a low-binding Durapore 0.22-μm-pore-size membrane filter (Ultrafree-MC; Millipore) to ensure complete removal of bacteria before removal from containment facilities.

Western blots were performed using rabbit anti-SigG at 1:1000 dilution and anti-rabbit conjugated to horseradish peroxidase (Dako), at a 1:5000 dilution, or using Penta-His HRP conjugated antibody at a 1:5000 dilution (Qiagen). The blot was developed using ECL Western blotting detection reagents (GE Healthcare).

For *β*-galactosidase activity, protein levels of cell-free extracts were quantified using a BCA kit (Pierce) and *β*-galactosidase activity was determined as described^14^ and expressed in Miller units per milligram of protein.^27^

### 2.4 RNA extraction

RNA was prepared from mycobacteria using the FastRNA Pro Blue kit (Qbiogene). Contaminating DNA was removed using RNase-free DNase (Roche), and RNA purified using RNeasy Minikit (QIAgen), or with a TURBO DNA-free kit (Ambion). RNA quality was determined using an Agilent Bioanalyser 2100 (Agilent Technologies).

### 2.5 Transcriptional start site mapping

Primer extension was carried out using Primer Extension System (Promega). Briefly, primer sigGPExt (Supplementary Table 2) was end-labelled with ^32^P-ATP and extension reactions carried out using 40–100 μg RNA. Reactions were run on an 8% denaturing polyacrylamide gel, alongside sequencing reactions (T7 Sequenase Kit Amersham), and visualised using autoradiography. The transcriptional start site of *Rv0887c* was mapped using the GeneRacer kit (Invitrogen) for RNA ligase-mediated rapid amplification of 5′ cDNA ends. Briefly the GeneRacer Oligo (Invitrogen) was ligated to the 5′-ends of RNA from *M. tuberculosis* H37Rv. cDNA products for the genes of interest were produced by RT-PCR using a primer specific to the GeneRacer Oligo along with a gene specific primer (Supplementary Table 2). Amplified cDNA ends were cloned into pCR 4-TOPO (Invitrogen) for sequencing.

### 2.6 Microarray

Whole genome *M. tuberculosis* microarray slides were obtained from the Bacterial Microarray Group at St. George's, London. Cy5-labelled RNA versus Cy3-labelled DNA hybridizations were performed and analysed as previously described.^19,39^ Significant differences were determined using two-way ANOVA with the Benjamini and Hockberg False Discovery Rate correction. Fully annotated microarray data have been deposited in BμG@Sbase (accession number TBC) and ArrayExpress (accession number TBC).

### 2.7 Quantitative RT-PCR

cDNA synthesis was performed using Superscript II (Invitrogen) and random hexamer primers. Real-time quantitative PCR was carried out using Fast SYBR green master mix (Applied Biosystems) on an Applied Biosystems 7500 Fast instrument and analysed with 7500 Fast SDS software version 1.4. Gene specific primers (see Supplementary Table 2) were designed using Primer Express version 3.0 (Applied Biosystems). For each gene cDNA samples (and their RT negative controls) were run along with a set of genomic DNA standards to give a quantity of specific cDNA per sample. Values for the RT negative controls were subtracted, followed by normalisation to the corresponding value of the housekeeping gene *rrs* encoding 16s rRNA or *sigA*, to give relative expression level. Where required the relative expression of each sample was divided by that of the untreated sample or empty vector control to give the induction ratio.

### 2.8 Murine bone marrow-derived macrophage (BMDM) infection model

Infection of BMDM was carried out as previously described.^20^ Monocytes were isolated from the hind legs of female 6–8 week old BALB/c mice, allowed to differentiate into macrophages for 6 days and flushed from Petri dishes as described previously.^43^ Macrophages were seeded into 24-well plates at a density of 2 × 10^5^ cells per well. To activate macrophages 10 ng ml^−1^ murine interferon-*γ* (IFN-*γ*; Roche) was added. Naïve and activated macrophages were infected with *M. tuberculosis* strains grown to mid-log (OD_600_ ∼ 0.5) and resuspended in PBS containing 0.05% Tween 80 at an MOI of 0.1:1. Survival and multiplication of *M. tuberculosis* was determined by calculating CFU's at time points post infection.

## 3 Results

### 3.1 Characterisation of the SigG operon

**Figure 1:**
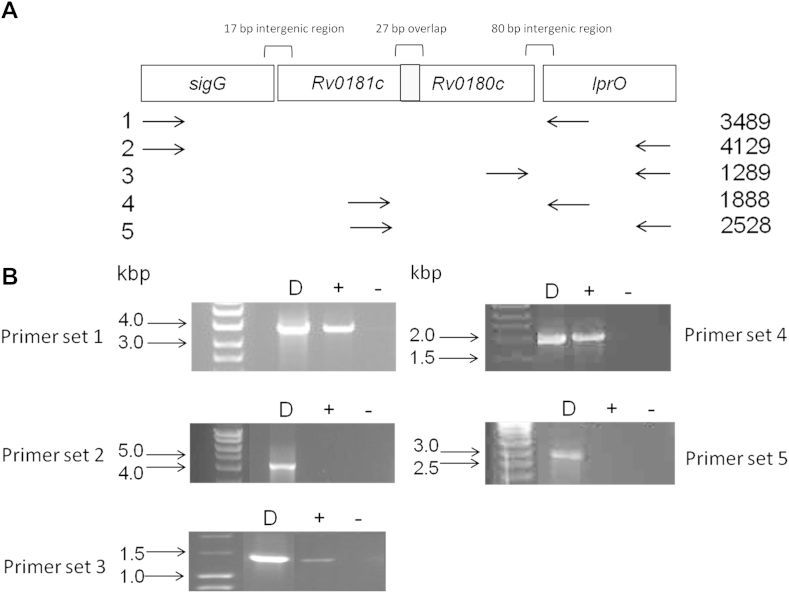
Co-transcription of *sigG*, *Rv0181c* and *Rv0180c* but not *lprO*. (A) Schematic representation of *M. tuberculosis* genomic region containing *Rv0182* (*sigG*)*-Rv0179c* (*lprO*). Includes positions of primers used in RT-PCR. (B) RT-PCR showing co-transcription of *sigG* operon. Transcript was detected using primer sets 1, 3 and 4 but not 2 and 5 indicating *sigG* transcript terminates within *lprO* between primer sets 1 and 2 and that *lprO* transcription starts within *Rv0180c*. + shows RT positive; − shows RT negative control; D shows genomic DNA PCR control; sizes of DNA markers are indicated.

As a first step in the characterisation of SigG, we confirmed its genetic organisation. The gene immediately upstream of *sigG* is transcribed in the opposite orientation, and downstream there are three genes transcribed in the same orientation (Figure 1A). Examination of correlated gene expression predicts that an operon runs from *sigG-Rv0180c*, missing *lprO* (Pearsons correlation coefficient 0.37 *sigG-Rv0180c*, 0.08 *sigG-lprO*, TB database www.tbdb.org). We confirmed this to be the case by RT-PCR (Figure 1) using a series of primers spanning from the beginning of *sigG* to the end of *lprO*. Transcripts could be obtained from *sigG* to primer lprOR1 located 250 bp within *lprO* as well as from the end of *Rv0181c* to lprOR1, but not to the 3′ end of *lprO* indicating that the operon terminates within *lprO*. A further transcript could be detected between the 3′ end of *lprO* and 300 bp upstream of the *Rv0180c* stop codon. Therefore, the *sigG* operon consists of *sigG*, *Rv0181c* and *Rv0180c*, while the *lprO* transcript starts from a promoter internal to *Rv0180c*.

### 3.2 Determining *sigG* transcriptional start sites

**Figure 2:**
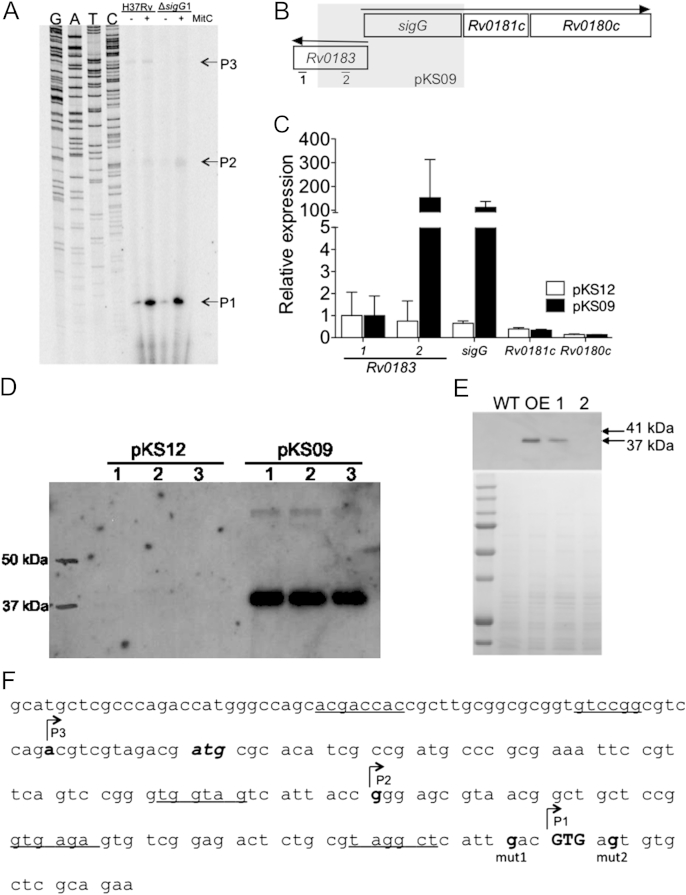
Transcriptional and translational analysis of *sigG*. (A) Primer extension analysis using primer sigGPExt, located within the *sigG* annotated coding region. RNA was isolated from wild-type *M. tuberculosis* H37Rv and Δ*sigG*1 with and without treatment with 0.02 μg ml^−1^ mitomycin C (MitC) for 16 h. (B) Schematic of *sigG* operon and *Rv0183* gene organisation showing region contained within pKS09 (SigG over expression construct, highlighted in grey) and position of *Rv0183* primers used in (C). (C) qRT-PCR showing expression of *sigG, Rv0181c, Rv0180c* and *Rv0183* from primers located outside (1) or within (2) pKS09 in wild-type H37Rv containing the SigG over-expression construct (pKS09) or empty vector (pKS12). Data shows expression normalised to *sigA*, mean + standard deviation for 3 biological replicates. (D) Western blot analysis using anti-SigG antibody of three colonies of wild-type H37Rv containing the SigG over-expression construct (pKS09) or empty vector (pKS12). Sizes of marker bands are indicated. (E) Translational start site assay. Western blot using anti-SigG antibody against cell free extracts from *M. smegmatis* containing no vector (WT), or *M. smegmatis* containing pKS09 SigG over-expression construct (OE), pKS09TTSmut1 (1) or pKS09TTSmut2 (2) translational start site frame shift mutations of SigG, deletions as indicated in (F). Equal loading was verified by Coomassie Blue staining of a similar gel (shown below Western blot). The expected sizes for SigG starting at the annotated start site (41 kDa), or the experimentally determined start site (37 kDa) are indicated. (F) DNA sequence of promoter region of *sigG* showing position of transcriptional start sites identified in (A), P1, P2 and P3 with potential promoter regions underlined, annotated start codon *bold italics*, experimentally determined start codon BOLD CAPITALS, and location of single base pair deletions used in (D) mut1 and mut2.

To determine potential promoters for the *sigG* operon the transcript start sites were mapped by primer extension using RNA extracted from wild-type *M. tuberculosis* H37Rv and Δ*sigG*1 strains grown with and without mitomycin C treatment (Figure 2A). This revealed three potential start sites located at −12, +57 and +114 bp from the annotated start codon of *sigG*, labelled P3, P2 and P1 respectively. P1 shows induction by the DNA damaging agent, mitomycin C, and also gives the strongest band both with and without mitomycin C treatment. P2 and P3 give much weaker bands with P2 showing some induction by mitomycin C and P3 being potentially SigG dependent, being only identified from wild-type RNA.

### 3.3 Mapping SigG translational start site

As both P1 and P2 are located downstream of the annotated translational start site of *sigG*, this site must be incorrect. Located immediately downstream of P1 are 2 potential GTG start codons (Figure 2F). To determine if one of these is the start codon for *sigG* we performed a frame-shift mutagenesis translational start site assay as previously described.^40^ Due to the low expression of SigG during normal growth^25^ protein could not be detected. Instead a SigG over-expression construct, pKS09, was developed. This plasmid was derived from the moderate copy number plasmid pMV261^44^ and contains the entire *sigG* annotated coding region plus 500 bp upstream, to include its own regulatory elements (see Supplementary Table 1). This region also contains a significant proportion of the upstream gene *Rv0183* (Figure 2B). Quantitative RT-PCR analysis found substantial over-expression of *sigG* when transformed into wild-type *M. tuberculosis* H37Rv. Expression of *Rv0181c* and *Rv0180c* was unaffected in this strain (Figure 2C), indicating that the genomic *sigG* operon was not over-expressed. Further RT-PCR analysis found that this transcript started within the vector sequence (data not shown) indicating that *sigG* over-expression was due to read-through from a vector encoded promoter and not auto-regulation by SigG. Western blot on cell free extract obtained from this strain, using polyclonal antibody against *M. tuberculosis* SigG, found that the expressed protein was 4 kDa smaller than expected from the annotation (Figure 2D). Two individual single base pair deletions were made in this construct immediately up or downstream of the 1st potential GTG start codon downstream of P1, termed pKS09TTSmut1 and pKS09TTSmut2 respectively (Figure 2F). These constructs were expressed in *M. smegmatis*, which has been shown to be a suitable model for determining translational start codons of *M. tuberculosis* proteins.^40^ Western blots were performed on cell-free extracts obtained from these strains grown to mid-log phase, and equal loading was determined by Coomassie staining of an equivalent gel (Figure 2E). SigG was undetectable in wild-type *M. smegmatis*, whereas the over-expression construct gave high levels of SigG protein. SigG could be detected in pKS09TTSmut1 but was not detectable with pKS09TTSmut2. This indicates that the translational start site for SigG is between these two mutations and so must be located at the GTG 114 bp downstream of the annotated start codon resulting in a protein 38 amino acids shorter.

### 3.4 Promoter identification for *sigG*

**Figure 3:**
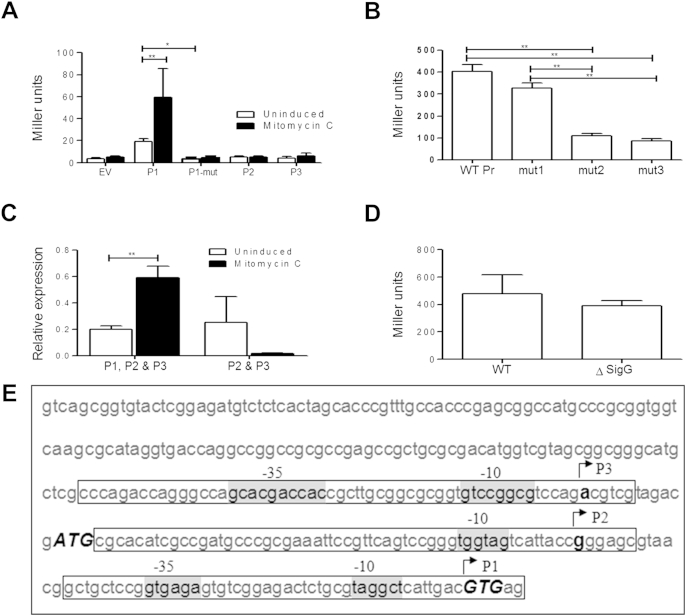
Promoter activity for *sigG*. (A) β-galactosidase activity for *M. tuberculosis* containing pEJ414 empty vector (EV), or vectors containing translational fusions of each promoter identified in Figure 2A to reporter gene *lacZ* (as indicated in E), pLDlac1 (P1), pLDlac2 (P2) and pLDlac3 (P3) and P1 containing a mutation of the −10 region (pLDlac1-mut, P1-mut) with (black bars) and without (white bars) treatment with 0.02 μg ml^−1^ mitomycin C. (B) Translational fusion of 339 bp region upstream of *sigG* containing all 3 putative promoters (pAG04, WT Pr) with mutations to −10 regions of P1 (pAG04-mut1), P1 and P2 (pAG04-mut2) and P1, P2 and P3 (pAG04-mut3). (C) qRT-PCR using primers sigGqRTF and sigGqRTR (located downstream from P1, detecting transcript from all promoters), and sigGP2qRTF and sigGP2qRTR (located between transcriptional starts sites P1 and P2, detecting transcript starting upstream of P1). Expression was assessed from wild-type *M. tuberculosis* H37Rv with (black bars) and without (white bars) mitomycin C treatment and normalised to *sigA*. (D) β-galactosidase activity for pAG04, containing the full length *sigG* promoter in wild-type *M. tuberculosis* H37Rv and Δ*sigG*WO. (E) 339 bp DNA sequence of promoter region of *sigG* used to make construct pAG04 including P1, P2 and P3 with potential −10 and −35 promoter regions shaded grey. Locations of the DNA fragments used in the individual pLDlac promoter constructs are indicated by boxes, together with the transcriptional start site for each promoter (arrow and bold) and the annotated (ATG) and experimentally determined (GTG) start codons *BOLD ITALIC CAPITALS*. Data represents mean + standard deviation from 3 biological replicates, statistical significance by two-tailed *T* test (*P* value * < 0.1, ** < 0.01).

SigG translation starts immediately downstream of P1, which corresponds with the previously identified RecA_NDp promoter^21^ (Figure 2F). To determine whether each of the potential promoters were active, transcriptional fusions for each promoter were constructed fusing approximately 60 bp upstream of each transcriptional start site to the reporter gene *lacZ*, giving plasmids pLDlac1, pLDlac2 and pLDlac3 (containing P1, P2 and P3 respectively, Figure 3E). The promoter activity for each construct was determined in wild-type *M. tuberculosis* H37Rv with and without mitomycin C treatment (Figure 3A). Only P1 gave activity above background and this was increased approximately 3-fold upon DNA damage induction. An A to C mutation was made in the −10 region of RecA_NDP of P1 giving pLDlac1-mut. This mutation is known to inactivate this promoter family^22^ and abolished the activity from the P1 promoter. Therefore, promoter 1 is active and is responsible for DNA damage induction of the *sigG* operon. To determine whether the other potential promoters play a role in expression of *sigG*, pGA04 was constructed, containing all 3 potential promoters fused to the reporter gene *lacZ* (Figure 3E). This longer construct could also contain binding sites for other potential regulatory elements necessary for activity that may be missing in the smaller constructs. Mutations were made in the −10 region of P1 (as above) giving pAG04-mut1, and also in the −10 regions of P2 and P3 giving plasmids, pAG04-mut2 (P1 and P2 mutation) and pAG04-mut3 (P1, P2 and P3 mutation). The promoter activity for these plasmids was determined in wild-type *M. tuberculosis* H37Rv (Figure 3B). Mutation of the RecA_NDp (pAG04-mut1) reduced but did not abolish activity from the wild-type promoter (pAG04); while this was not a significant reduction this may be due to the presence or absence of additional regulatory elements of P1 in the longer construct. Additional mutation of the −10 region of P2, (giving pAG04-mut2), reduced this activity further, to approximately 1 quarter that of the wild-type, whereas mutation of P3 had no additional effect. Therefore *sigG* is expressed from at least two promoters. To confirm this at the mRNA level qRT-PCR was performed on RNA isolated from *M. tuberculosis* H37Rv with and without mitomycin C treatment (Figure 3C). Primers were located either downstream of promoter P1, therefore detecting transcripts originating from all three promoters, or between promoter P2 and P1, therefore detecting transcripts originating from promoters P3 and P2 only. Detection of transcripts downstream of P1 showed a high level of expression and was induced 3-fold upon mitomycin C induction. A similar level of transcript could be detected upstream using primers located between P1 and P2 but outside of the *Rv0183* coding region. This transcript reduced upon DNA damage induction. The difference between the transcripts detected by the two primer sets is most likely directly due to the activity of promoter P1. Therefore under DNA damaging conditions P1 is the primary promoter for the *sigG* operon. One other promoter possibly also controls expression of *sigG*, however, due to the proximity of the *Rv0183 and sigG* coding regions, the *sigG*P2qRTF primer is located only 37 bp upstream of the annotated *Rv0183* coding region. It is therefore possible that the transcript detected upstream of *sigG* promoter P1 is actually the *Rv0183* transcript.

Primer extension analysis indicated that P3 was potentially SigG dependent (Figure 2A); however, over-expression of SigG did not affect expression from the rest of the *sigG* operon, suggesting that expression of the genomic operon is not SigG dependent (Figure 2C). Therefore we examined activity of the SigG promoter region in Δ*sigG*WO, in which the *SigG-Rv0180c* operon was deleted and replaced with a gentamicin resistance cassette by homologous recombination (Supplementary Figure 1A). The first 32 bp of *sigG* and last 380 bp of *Rv0183* were included in the targeting construct to prevent effects on the promoters of adjacent genes. The absence of expression of *sigG, Rv0181c* and *Rv0180c* in Δ*sigG*WO was confirmed by qRT-PCR (Supplementary Figure 1B). The *sigG* promoter construct pAG04, containing all three potential promoters was expressed in Δ*sigG*WO, but showed no difference in activity compared to expression in wild-type *M. tuberculosis* (Figure 3D). The dominant P1 promoter could be masking the effects of a *sigG* dependent promoter. However, it is possible that lack of detection of the P3 transcriptional start site in Δ*sigG*1 is due to a defect in this strain, which also contains a spontaneous mutation affecting the cell wall.^39^

### 3.5 Investigation of the role of SigG *in vivo*

**Figure 4:**
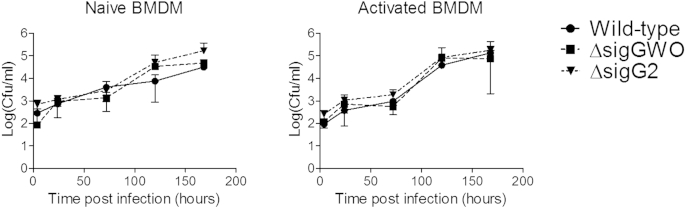
SigG does not affect survival of *M. tuberculosis* during infection of macrophages. Survival and multiplication of *M. tuberculosis* H37Rv, mutants ΔsigG2 and ΔsigGWO in naïve and activated BMDMs. Data represents the mean + standard deviation of 3 biological replicates.

The potential role of SigG during *in vivo* infection was assessed in a macrophage infection model. Wild-type H37Rv, Δ*sigG*2 and Δ*sigG*WO, were used to infect naïve and activated murine bone marrow derived macrophages (BMDM). Neither the Δ*sigG*2 or Δ*sigG*WO strains were attenuated compared to wild-type (Figure 4).

### 3.6 Identification of the SigG regulon

The possible function of SigG was further assessed by determining its regulon. Previous investigations examining the gene expression profiles of a *sigG* mutant strain with and without DNA damage induction found no significant differences from the wild-type H37Rv strain.^39^ Therefore, in this investigation we compared the expression profiles of *M. tuberculosis* H37Rv containing pKS09, a SigG over-expression construct, to that of H37Rv containing an empty vector (pKS12) by microarray (Table 1). This identified 13 genes significantly up-regulated in the over-expression strain by more than 1.75 fold (*P* value < 0.05). The two most upregulated genes were *sigG* and *Rv0183*. A significant portion of *Rv0183* is located within the pKS09 construct used for over-expression (see Figure 2B). Quantitative-PCR was performed using two sets of primers, one located either within *Rv0183* and contained in pKS09 (primer set 2), the other within *Rv0183* but outside of the region contained in pKS09 (primer set 1). This confirmed that the higher level of *Rv0183* RNA observed in the microarray analysis is an artefact of the over-expression strain and not specific regulation by SigG (Figure 2C). *Rv0181c* and *Rv0180c* were not identified as being upregulated in pKS09, which confirmed previous qRT-PCR analysis (Figure 2C) and shows further shows that SigG is not autoregulatory. Quantitative PCR confirmed that 9 of the remaining 11 genes were upregulated more than 1.5-fold compared to an empty vector strain (Table 1). To confirm whether these genes identified by microarray analysis were upregulated due specifically to the SigG protein, or artefacts of the over-expression construct, induction relative to the empty vector containing strain was assessed in H37Rv containing pKS09FS, in which a single base pair deletion was made within the coding region of *sigG*. This resulted in a strain which gave the same level of RNA but no functional SigG protein due to a frame-shift (Table 1). Induction of *Rv0942, vapB15, vapC15, gabD1* and *Rv2004* occurred in both the SigG overexpression (pKS09) and frame-shift (pKS09FS) strains and so most likely represent false positives. The *vapBC15* operon encodes a toxin-anti-toxin system, the toxin of which degrades mRNA and has been shown to be induced during stress such as hypoxia.^33^ This operon may be instead responding to the abnormal pattern of RNA expression rather than to SigG itself. Induction of *fprB, Rv0887c, Rv0911, Rv0912, prpD* and *inhA* was significantly reduced in the SigG frame-shift (pKS09FS) compared to functional SigG overexpression (pKS09) indicating that their induction was dependent on functional SigG protein.

**Figure 5:**
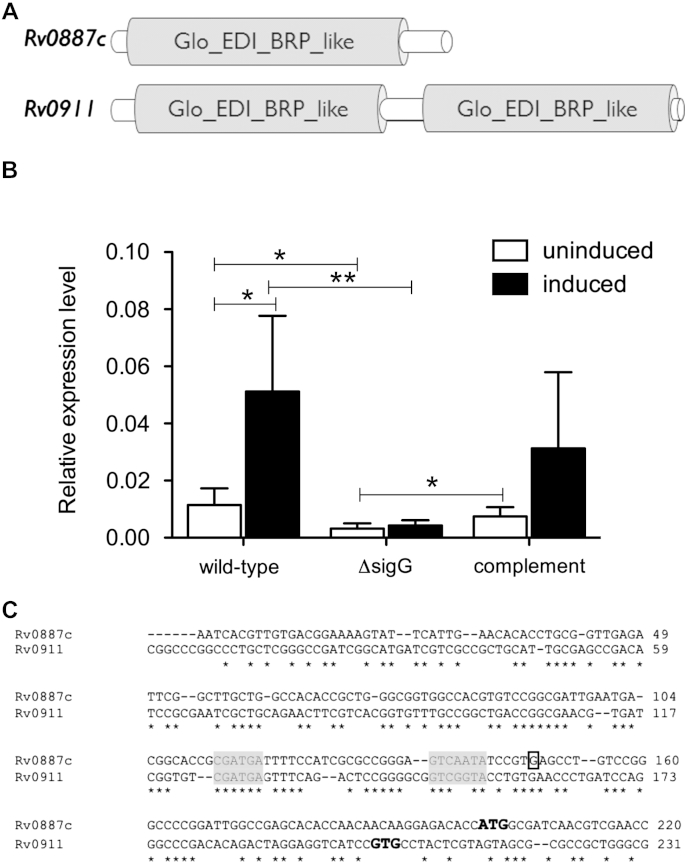
SigG regulates 2 potential glyoxylases, Rv0887c and Rv0911. (A) Domain structure of Rv0887c and Rv0911 showing position of Glo_EDI_BRP_like domains. (B) Expression of *Rv0887c* analysed by qRT-PCR in wild-type H37Rv, Δ*sigG2* mutant and Δ*sigG2* complement (containing pLDL-8T) with (black bars) and without (white bars) mitomycin C induction. Data shows expression normalised to *rrs*, mean + standard deviation for at least 5 biological replicates, statistical significance by two-tailed *T* test (*P* value * < 0.1, ** < 0.01 and *** < 0.001). (C) 5′RACE of *Rv0887c* yielded a transcriptional start site, which mapped to a G residue 52 bp upstream of the annotated coding region (boxed). CLUSTAL 2.1 alignment of the upstream regions of *Rv0887c* and *Rv0911* showed that this G residue was conserved 40 bp upstream of the annotated start codon of Rv0911 and was located in a region of strong homology between the two genes. A *sigG* promoter sequence CGATGA(N_18_)GTCNNTA was predicted (grey shading) from the alignment. Annotated start codons are shown in bold.

The two genes most highly induced by SigG were *Rv0887c* (16-fold induction) and *Rv0911* (11-fold induction). These are both annotated as conserved hypothetical proteins of unknown function, however, analysis of their sequence structure identified that they both contain Glo_EDI_BRP_like superfamily domains (Figure 5A). These domains are characteristic of glyoxalases, type I extradiol dioxygenases and bleomycin resistance proteins, which are all involved in inactivation of toxic compounds, many of which damage DNA.^18^ Expression of *Rv0887c* in response to DNA damage by mitomycin C was assessed in wild-type H37Rv and Δ*sigG*2, in which *sigG* is mutated, without the spontaneous cell wall mutation^39^ and Δ*sigG*2 containing the pLDL-8T complementing vector, containing the entire *sigG* operon (Figure 5B). Expression of *Rv0887c* was found to increase upon mitomycin C treatment in the wild-type H37Rv strain (induction ratio 5.7, *p*-value <0.05). Expression in the Δ*sigG*2 mutant was 3.6-fold lower than wild-type without induction, and no increase in expression was seen after treatment with mitomycin C (induction ratio 1.5, *p*-value > 0.1). Expression in the complementing strain was similar to wild-type (induction ratio 3.9, *p*-value < 0.05). There was also a slight, but not significant, increase in expression of *Rv0911* with mitomycin C treatment in wild-type H37Rv, which was reduced in the mutant (data not shown). This indicates that expression and DNA damage induction of *Rv0887c* is dependent on SigG.

The transcriptional start site of *Rv0887c* was mapped using 5’ RACE in wild-type H37Rv expressing pKS09. This identified a potential start site 52 bp upstream of the annotated start codon (Figure 5C). This region showed significant homology to the promoter region of *Rv0911* and was used to identify a potential promoter motif for SigG as being CGATGA(N_18_)GTCNNTA.

## 4 Discussion

We examined the regulation of *sigG* at the transcriptional level, as well as determining the regulon for SigG. The dominant promoter for *sigG* was found to coincide with the RecA_NDp promoter. This promoter is located downstream of the annotated start site for *sigG* indicating that this site must be incorrect. Due to the GC rich genome incorrect annotation of translational start codons is common in Mycobacteria, which has implications for predictions of promoters and gene function.^40^ Translational start site mapping of SigG revealed that the start codon is in fact located 114 bp downstream of the annotated start codon, producing a protein 38 amino acids shorter. This region does not contain any of the functional domains for the sigma factor. Codon usage database analysis^30^ found that this region contained 7 (18.4%) codons which occur at a frequency less than 5 per 1000 for the entire *M. tuberculosis* protein coding genes, compared to 15 (4.5%) in the rest of the *sigG* gene. This may mean that this region is much less able to be translated. The alternative sigma factor, SigE, was shown to start from three different codons depending on the promoter used,^16^ it is therefore possible that SigG is expressed from the annotated start codon under different growth conditions. The DNA damage inducible promoter for SigG is the furthest downstream, therefore if SigG was also able to start from two translational start codons you would expect the protein expressed under DNA damaging conditions to be smaller than that expressed under normal, exponential growth. In fact there was no evidence of any larger isoforms from over-expression of SigG in either *M. smegmatis* or *M. tuberculosis* (Figure 2). It was also noted that expression of soluble recombinant SigG in *E. coli* could only be obtained when this region was omitted from the expression construct (data not shown). This indicates that SigG likely starts from this site under all conditions.

This study identified three potential promoters upstream of the experimentally determined SigG translational start site, including the previously characterised DNA damage inducible RecA_NDp, recognised by ClpR.^21,45^ Expression of sigma factors from multiple promoters has been shown previously for SigB, SigC, SigE and SigL.^11,13,38^ We were able to demonstrate the activity and DNA-damage inducible nature of promoter P1; however, when included in the longer *lacZ* construct, mutation of the P1 −10 region did not significantly reduce expression levels. This is likely due to the presence of regulatory elements in this longer construct that were not present in the individual promoter constructs. We were unable to ascertain whether promoters 2 and 3 were genuine; both promoters showed no activity when expressed individually, and the longer construct, containing potential binding sites for other regulatory elements, showed only a slight reduction in activity when promoter 2 was mutated. It's possible that the mutations used did not completely eliminate activity or an additional, uncharacterised promoter is responsible for the residual activity. Initial primer extension analysis indicated that promoter 3 was SigG dependent, however this is unlikely to be the case as over-expression of SigG had no effect on expression from its own promoter (as expression of the rest of the operon was unaffected); also promoter activity was unaffected in a SigG mutant strain (Figure 3D). Promoter 3 does, however, bear some similarity to the SigM consensus, which has been shown to be induced during temperature stress and stationary phase and regulate genes involved in oxidative stress and secretion.^2,4,35^

*sigG* was found to be transcribed in a three gene operon with a long 3′ untranslated region overlapping the downstream gene *lprO*. Whole genome sequencing has revealed that long 3′ UTR's are common in *M. tuberculosis*.^3^ In most bacteria termination is tightly regulated by a hairpin loop structure followed by a poly-U-stretch, conversely mycobacterial terminators lack the poly-U-stretch which may result in less efficient termination and have implications for the regulation of downstream genes.^3,28^ Interestingly, when the entire *sigG* operon, up to 380 bp before the end of *Rv0180c*, was deleted an increase in the expression of *lprO* was observed (Supplementary Figure 1), indicating that during normal growth either expression of the *sigG* operon inhibits *lprO* expression or a negative regulatory element for *lpr*O is present within the deleted section of *Rv0180c*.

SigG is regulated as part of the DNA damage response of *M. tuberculosis.* However, mutation of *sigG* caused no increase in sensitivity to DNA damaging agents.^39^ SigG was predicted to regulate the RecA-dependent SOS response, in particular the SOS response regulator *lexA.*^24^ Subsequent investigations found no difference in the regulation either the SOS response, or RecA-independent DNA damage response genes in a *sigG* deletion mutant, and *lexA* was found to be controlled by a SigA consensus promoter.^39^ Our previous analysis of the transcriptional profile of a *sigG* mutant strain in fact showed no significant differences to the wild-type *M. tuberculosis*.^39^ We, therefore, looked at genes upregulated with a *sigG* over-expression strain. This gave five genes that were upregulated dependent on functional SigG, as their expression was no longer increased when a frameshift was introduced into the SigG coding region: *fprB, Rv0887c, Rv0911, Rv0912*, *inhA* and *prpD*. In addition, *Rv0887c* expression was confirmed to be regulated by SigG as its expression and DNA damage induction was reduced in a *sigG* mutant strain. No genes previously predicted to be part of the SOS response or the RecA-independent DNA damage response were found to be upregulated in the SigG over-expression strain, further confirming that SigG does not regulate either of these pathways.

The two genes most highly upregulated in the SigG over-expression strain were *Rv0887c* and *Rv0911*. These encode conserved hypothetical proteins of unknown function that both contained glyoxylase-like domains. Glyoxylase is involved in detoxifying reactive 2-oxoaldehydes, mainly methylglyoxal, a by-product of glycolysis thought to control the rate of carbon flux when moving between environments.^18^ Methylglyoxal can also cause DNA damage. Rv0911 was predicted to be involved in the methylglyoxal detoxification pathway of *M. tuberculosis*, as it was able to interact with pyrimidine-imidazole compounds, which target the methylglyoxal detoxification pathway.^26^ Proline metabolism has also been predicted to play a role in methylglyoxal detoxification in *M. smegmatis*.^5^ Accordingly deletion of PruC, a transcriptional activator of proline metabolism, resulted in an increase in expression of five potential glyoxalases, including *MSMEG_5680* the homologue of *Rv0887c*, as well as *sigG* and other genes belonging to the RecA-independent DNA damage induction pathway. Increased levels of methylglyoxal were found in *M. tuberculosis* infected macrophages and granulomas,^32^ *sigG* was found to be upregulated in *M. tuberculosis* infected macrophages, and *Rv0180c*, cotranscribed with *sigG*, has been shown to be involved in host cell invasion.^8,10^ However, a *sigG* mutant was not found to be impaired in its ability to infect mice (data not shown) or macrophages derived from mouse bone marrow (Figure 4). A previous study did find slight attenuation of a *sigG* mutant during infection of macrophages.^24^ This may reflect differences in strains of *M. tuberculosis* (H37Rv or CDC1551) or cell lines (murine BMDM or murine macrophage J774A.1) used. There was also no difference in the susceptibility of a *sigG* mutant strain to DNA damaging agents,^39^ or methylglyoxal toxicity compared to wild-type (data not shown). This could be due to compensation by other sigma factors or glyoxalases. For example Rv0577 is a predicted glyoxalase that has been shown to effect T cells to produce a Th1 response and induce maturation of dendritic cells.^7,26^ Infection of most mice strains with *M. tuberculosis* does not result in the highly organised granulomas seen in human infection,^34,37^ and so the role of SigG may not be as important during infection of mice.

The transcriptional start site of *Rv0887c* was mapped to a G residue, 52 base pairs upstream of the annotated start codon. Alignment of the upstream regions of *Rv0887c* and *Rv0911* showed significant homology between the two and a putative SigG promoter sequence was identified as CGATGA(N_18_)GTCNNTA. Previously a SigG consensus sequence has been hypothesised to be GCGNGT(N_15–18_)CGANCA^24^ however, at least two of the genes, whose decreased expression in a *sigG* deletion mutant was used to suggest they were part of the SigG regulon, have subsequently been show to not be SigG regulated: *lexA*^39^ and *Rv0654* (data not shown). It is therefore unlikely that this consensus is correct.

Rv0181c, which is cotranscribed with SigG, may also have a detoxifying role. BLAST analysis revealed that this protein had significant homology to the *E. coli* YhhW pirin protein (data not shown). YhhW has been shown to break down the antioxidant, quercetin, and is therefore a detoxifying enzyme known as a quercetinase.^1^ Purified Rv0181c protein has been shown to have some quercetinase activity thus supporting its potential role as a detoxifying enzyme (Supplementary Figure 2). Quercetin is a member of a group of compounds called flavonoids, which have been shown to inhibit fatty acid and mycolic acid biosynthesis in *M. tuberculosis.*^6^ In addition the strain Δ*sigG*WO showed increased susceptibility to the presence of Tween 80 compared to the wild-type strain. This phenotype could be complemented by all three genes of the *sigG* operon but not by *sigG* on its own (Supplementary Figure 3). This suggests that the susceptibility of the Δ*sigG*WO strain to Tween 80 is due to the absence of either *Rv0181c* and/or *Rv0180c*. Due to the fact that Tween 80 acts as a surfactant and Rv0180c is an integral membrane protein^47^ we hypothesise that the Tween 80 phenotype may be due to the absence of the *Rv0180c* gene.

We have shown that *sigG* is expressed as part of a three gene operon and its expression is controlled by at least two promoters. SigG is induced as part of the RecA independent DNA damage response but is not the sigma factor which controls this response.^39^ Instead SigG induces an alternative regulon, possibly involved in detoxification of methylglyoxal. *sigG* is co-transcribed with a putative quercetinase and a transmembrane protein. The absence of one or both of these two members of the *sigG* operon causes *M. tuberculosis* to become susceptible to the presence of Tween 80. Taken together, the potential functions of genes co-transcribed with *sigG* and members of the SigG regulon, outline a novel aspect of the DNA damage and stress response in *M. tuberculosis,* which, as well as inducing genes involved in DNA repair, also induces, via SigG, genes possibly involved in detoxifying factors that could be responsible for causing DNA damage and cell stress.

## Ethical approval

Not required.

## Funding

This research was supported by the UK Medical Research Council (programme number U1175 32056).

## Competing interests

None declared.

## Figures and Tables

**Table 1:** Genes upregulated in SigG over-expression strain. Genes found to be upregulated in SigG over-expression strain microarray analysis. Direct regulation by SigG was confirmed by qRT-PCR. Genes positively regulated by functional SigG are shown in bold.

Rv number	Gene name	Microarray†		qRT-PCR‡		Protein function
		Fold-change	*p*-Value	pKS09	pKS09FS	
*Rv0182*	*sigG*	110.22	4.11 × 10^−08^	473.1	443.9	Alternative RNA polymerase sigma factor
*Rv0183*		52.52	2.81 × 10^−04^	ND	ND	Lysophospholipase
***Rv0887c***		**15.96**	**2.36** × **10**^**−06**^	**229.9**	1.87**	**Conserved hypothetical protein**
***Rv0911***		**11.37**	**3.13** × **10**^**−07**^	**12.13**	1.297*	**Conserved hypothetical protein**
***Rv0886***	***fprB***	**10.89**	**3.13** × **10**^**−07**^	**6.72**	1.68**	**NADPH:adrenodoxin oxidoreductase**
***Rv0912***		**6.45**	**4.63** × **10**^**−04**^	**2.22**	1.04**	**Conserved transmembrane protein**
***Rv1130***	***prpD***	**1.93**	**0.042**	**2.44**	0.58*	**Methylcitrate dehydratase**
***Rv1484***	***inhA***	**1.78**	**0.0073**	**1.96**	1.05**	**NADH-dependent enoyl-[acyl-carrier-protein] reductase**
*Rv0942*		4.93	0.0014	1.22	1.04	Hypothetical protein
*Rv0234c*	*gabD1*	2.05	0.019	1.64	1.45	Succinate-semialdehyde dehydrogenase
*Rv2004c*		1.98	0.027	0.46	0.64	Conserved hypothetical protein
*Rv2009*	*vapB15*	1.98	0.027	2.81	2.45	Anti-toxin
*Rv2010*	*vapC15*	1.93	0.022	2.32	1.98	Toxin

† Microarray analysis performed with three biological and two technical replicates; fold change compared to empty vector containing wild-type *M. tuberculosis* H37Rv; genes upregulated >1.75 fold with Benjamini Hochberg corrected *p*-value < 0.05.

‡ qRT-PCT from at least three biological replicates; relative expression normalised to that of *sigA*; fold-change for SigG over-expression (pKS09), or SigG frameshift mutant (pKS09FS) compared to empty vector containing wild-type *M. tuberculosis*; significant differences between expression in pKS09 compared to pKS09FS containing strains determined by *T*-test (*p* < 0.1 = *; *p* < 0.01 = **).
