# Supplementary material for: Characterisation of the Mycobacterium tuberculosis alternative sigma factor SigG: Its operon and regulon
Source: Tuberculosis (Edinb). 2013 Sep;93(5):482–91. doi: 10.1016/j.tube.2013.05.005 (PMC3776920; doi:10.1016/j.tube.2013.05.005)
Supplement: Supplementary file 1 [file mmc1.docx]

**SUPPLEMENTARY DATA.**

**SUPPLEMENTARY MATERIALS AND METHODS**

**Quercetinase activity assay.** Quercetinase activity of His-Rv0181c was assessed as previously described[^1^](#_ENREF_1). Briefly the quercetin 2,3-dioxygenase activities of recombinant His-Rv0181c were assayed at room temperature for 5 min in a reaction mixture containing 50 mM NaH_2_PO_4_, pH 8.0, 300 mM NaCl, 60 μM quercetin in Me_2_SO, and 18.5 nM enzyme. Activity was observed by following the decrease in the absorbance maximum for quercetin, which occurs at 384 nm at pH 8.0.

**Alamar blue cell viability assay.** Antimicrobial susceptibility testing was performed as previously described[^2^](#_ENREF_2). *M. tuberculosis* strains were cultured to mid-log (OD_600_ 0.5) and inoculated into 96-well plates containing a two-fold serial dilution of Tween 80 at a bacterial concentration of 1 x 10^5^ cell per well. Wells containing Tween 80 only were used to detect auto-ﬂuorescence of compounds. Plates were incubated at 37°C for 10 days after which, 0.2 volumes Alamar blue reagent (CellTiter-blue, Promega) was added to each well and plates were incubated for a further 16 hours at 37°C. Fluorescence units (FU) were measured in a Cytoﬂuor II microplate ﬂuorometer (BMG Labtech) with excitation at 530 nm and emission at 590 nm. Percentage inhibition was defined as 1-(test well FU/mean FU bacteria only control) x 100.

**Supplementary Table 1. Plasmids used in this study. ***SDM: site directed mutagenesis.

| **Plasmid** | **Description*** | **Reference or source** |
| --- | --- | --- |
| pET28a | *E. coli* protein expression vector | Novagen |
| pEJ414 | Integrating mycobacterial *lacZ* transcriptional reporter vector | [^3^](#_ENREF_3) |
| pLDlac1 | pEJ414 containing 50 bp region containing putative P1 promoter made using primers sigGP1F and sigGP1R | This study |
| pLDlac1mut | pLDlac1 containing A to C mutation in -10 made by SDM using primers P1SDMF and P1SDMR | This study |
| pLDlac2 | pEJ414 containing 60 bp region containing putative P2 promoter made using primers sigGP2F and sigGP2R | This study |
| pLDlac3 | pEJ414 containing 60 bp region containing putative P3 promoter made using primers sigGP3F and sigGP3R | This study |
| pAG04 | pEJ414 containing 335 bp region containing putative P1, P2 and P3 promoter made using primers sigGPrF and sigGPrR | This study |
| pAG04mut1 | pAG04 containing A to C mutation in -10 of P1 made by SDM using primers P1SDMF and P1SDMR | This study |
| pAG04mut2 | pAG04mut1 containing GTA to TGC mutation in -10 of P2 by SDM using primers P2SDMF and P2SDMR | This study |
| pAG04mut3 | pAG04mut2 containing CGG to TCT mutation in -10 of P3 by SDM using primers P3SDMF and P3SDMR | This study |
| pMV261 | Mycobacterial expression vector containing *hsp60* promoter between XbaI site and multiple cloning site | [^6^](#_ENREF_6) |
| pKS12 | pMV261 with *hsp60* promoter removed by digest with XbaI and HindIII restriction sites | This study |
| pKS09 | pMV261 containing *sigG* annotated coding region and 619 bp upstream made using sigGxbaIF and sigGHindIIIR primers, which remove *hsp60* promoter | This study |
| pKS09TTS1 | pKS09 with single base pair deletion made by SDM using primers sigGTTS1SDMF and sigGTTS1SDMR | This study |
| pKS09TTS2 | pKS09 with single base pair deletion made by SDM using primers sigGTTS2SDMF and sigGTTS2SDMR | This study |
| pKS09FS | pKS09 with single base pair deletion made by SDM using primers sigGFSSDMF and sigGFSSDMR | This study |
| pMV306 | Intergrating Mycobacterial cloning vector | [^6^](#_ENREF_6) |
| pKP186 | pMV306 with intergrase removed | [^4^](#_ENREF_4) |
| pBS-INT | Mycobacterial suicide vector containing integrase, transformed in conjunction with pKP186 and its derivatives | [^5^](#_ENREF_5) |
| pLDL-8T | pKP186 containing sigG operon and 619 bp upstream of sigG start codon using primers sigGupXbaIF and sigGdownHindIIIR | This Study |

**Supplementary Table 2. Primers.**

| **Primer** | **Sequence** |
| --- | --- |
| sigGNheIF | GTGCTAGCGTGAGTGTGCTCGCAGAAAAC |
| sigGsiteR | CTCGAGTCACAGCGAATCGGGCAGGCC |
| sigGP1F | GCTGCTCCGGTGAGAGTGTCGGAGACTCTGCGTAGGCTCATTGACGTGAG |
| sigGP1R | CTCACGTCAATGAGCCTACGCAGAGTCTCCGACACTCTCACCGGAGCAGC |
| P1SDMF | CGGAGACTCTGCGTCGGCTCATTAAGC |
| P1SDMR | GCTTAATGAGCCGACGCAGAGTCTCCG |
| sigGP2F | CGCACATCGCCGATGCCCGCGAAATTCCGTTCAGTCCGGGTGGTAGTCATTACCGGGAGC |
| sigGP2R | GCTCCCGGTAATGACTACCACCCGGACTGAACGGAATTTCGCGGGCATCGGCGATGTGCG |
| sigGP3F | CCCAGACCATGGGCCAGCACGACCACCGCTTGCGGCGCGGTGTCCGGCGTCCAGACGTCG |
| sigGP3R | CGACGTCTGGACGCCGGACACCGCGCCGCAAGCGGTGGTCGTGCTGGCCCATGGTCTGGG |
| sigGPrF | TTTCTAGAGTCAGCGGTGTACTCGGAG |
| sigGPrR | GGAAGCTTCTCACGTCAATGAGCCTAC |
| P2SDMF | CCGTTCAGTCCGGGTGTGCGTCATTACCGGGAGCG |
| P2SDMR | CGCTCCCGGTAATGACGCACACCCGGACTGAACGG |
| P3SDMF | GCTTGCGGCGCGGTGTCTCTCGTCCAGACGTCG |
| P3SDMR | CGACGTCTGGACGAGAGACACCGCGCCGCAAGC |
| sigGXbaIF | ATATCTAGAGAGTGGGTCGGTGTTGTAAGC |
| sigGHindIIIR | ATAAAGCTTTCACAGCGAATCGGGCAGGC |
| sigGTTS1SDMF | CGTAGGCTCATTACGTGAGTGTGCTCGC |
| sigGTTS1SDMR | GCGAGCACACTCACGTAATGAGCCTACG |
| sigGTTS2SDMF | GGCTCATTGACGTGATGTGCTCGCAGAAAACTC |
| sigGTTS2SDMR | GAGTTTTCTGCGAGCACATCACGTCAATGAGCC |
| sigGFSSDMF | GCCCACACCGACCCTACCGGCG |
| sigGFSSDMR | CGCCGGTAGGGTCGGTGTGGGC |
| sigGupXbaIF | GCTCTAGAGAGTGGGTCGGTGTTGTAAGCCTGGAC |
| sigGdownHindIIIR | CCCAAGCTTATAGGATGACCGCCGCCGAAGTTGTA |
| RTsigGF | TTTGGATCCGTGAGTGTGCTCGCAG |
| RTRv0180c3’ | AGCCGTACCAGCGTTCTTCC |
| *lpr*OR1 | TGGCCGCCGTTGTTCATCTC |
| *lpr*OR2 | GTCCGGGGTGTAGTTGC |
| sigGPext | TCGGTGTGGGCGGAGAAGTC |
| GeneRacer RNA 5’ Oligo (Invitrogen) | CGACTGGAGCACGAGGACACTGA |
| RACE887 | GCCCCAGTTCGACGGCGTCAAAG |
| **qRT-PCR primers** | |
| sigAqRTF | TCGGTTCGCGCCTACCT |
| sigAqRTR | TGGCTAGCTCGACCTCTTCCT |
| rrsqRTF | AAGAAGCACCGGCCAACTAC |
| rrsqRTR | TCGCTCCTCAGCGTCAGTTA |
| sigGqRTF | TGAACTGCTCGCACACTGCTA |
| sigGqRTR | AGCGTCTCCTGAACAAGGTCTT |
| Rv0181cqRTF | CTGGCCCAACGCATGTC |
| Rv0181cqRTR | TCGGTAGCAGAATCGTTCATTTC |
| Rv0180cqRTF | TGGCCGCGCTCTATCTG |
| Rv0180cqRTR | AACGCGATTGGGAAATGC |
| lprOqRTF | TGCTCGCCCAAGCGATAG |
| lprOqRTR | CAGTGACCACCTGCGTTGAG |
| sigGP2qRTF | GGAGACTCTGCGTAGGCTCATT |
| sigGP2qRTR | TCGCGGCCAGAGTTTTCT |
| Rv0183_1qRTF | TGCACGGCACCGATGAC |
| Rv0183_1qRTR | GCCGATCCCACACATTCG |
| Rv0183_2qRTF | TGCTAGTGAGAGACATCTCCGAGTA |
| Rv0183_2qRTR | CTTGCACCCGGGATATTCC |
| Rv0887cqRTF | ACGCATCAACGGCTTTACG |
| Rv0887cqRTR | TTGCCGCCGCACATC |
| Rv0911qRTF | TGGCGCACCGAACAGAT |
| Rv0911qRTR | GCTGATCGTCGAACCATGCT |
| fprBqRTF | GCTGATCGTCGAACCATGCT |
| fprBqRTR | GGGCCGCTTCGGGTAAT |
| Rv0912qRTF | GCACTCACGGTGTGGTACTACAA |
| Rv0912qRTR | CACCGAAGTACAGCCCGTATTC |
| Rv0942qRTF | TGTCCCGCCAATAGCTTGAG |
| Rv0942qRTR | CGATCATCAAAAGATAGGGTTTCTG |
| gabD1qRTF | CCCATCGCCACCATCAAC |
| gabD1qRTF | CCGCGTCGACTTCGTCAT |
| Rv2004cqRTF | GGATTGCTTGGAGTTCGAAGAC |
| Rv2004qRTR | CAGATCCATGGCCAAAAAGG |
| vapB15qRTF | TCACGGACCAACATCGAGATC |
| vapB15qRTR | GCTTGGAATCGAGTCGGTACA |
| vapC15qRTF | GCTAGCCGATCGCATTGC |
| vapC15qRTR | CTTACCGATCAGCAGCTCCAT |
| prpDqRTF | TGAAGTGATCGTGGACGAACTG |
| prpDqRTR | CTGTTCAACGGGTTCCACTACA |
| inhAqRTF | TGGGCATCAACCCGTTCT |
| inhAqRTR | GAGATGTGGATGCCCTTGGA |

**
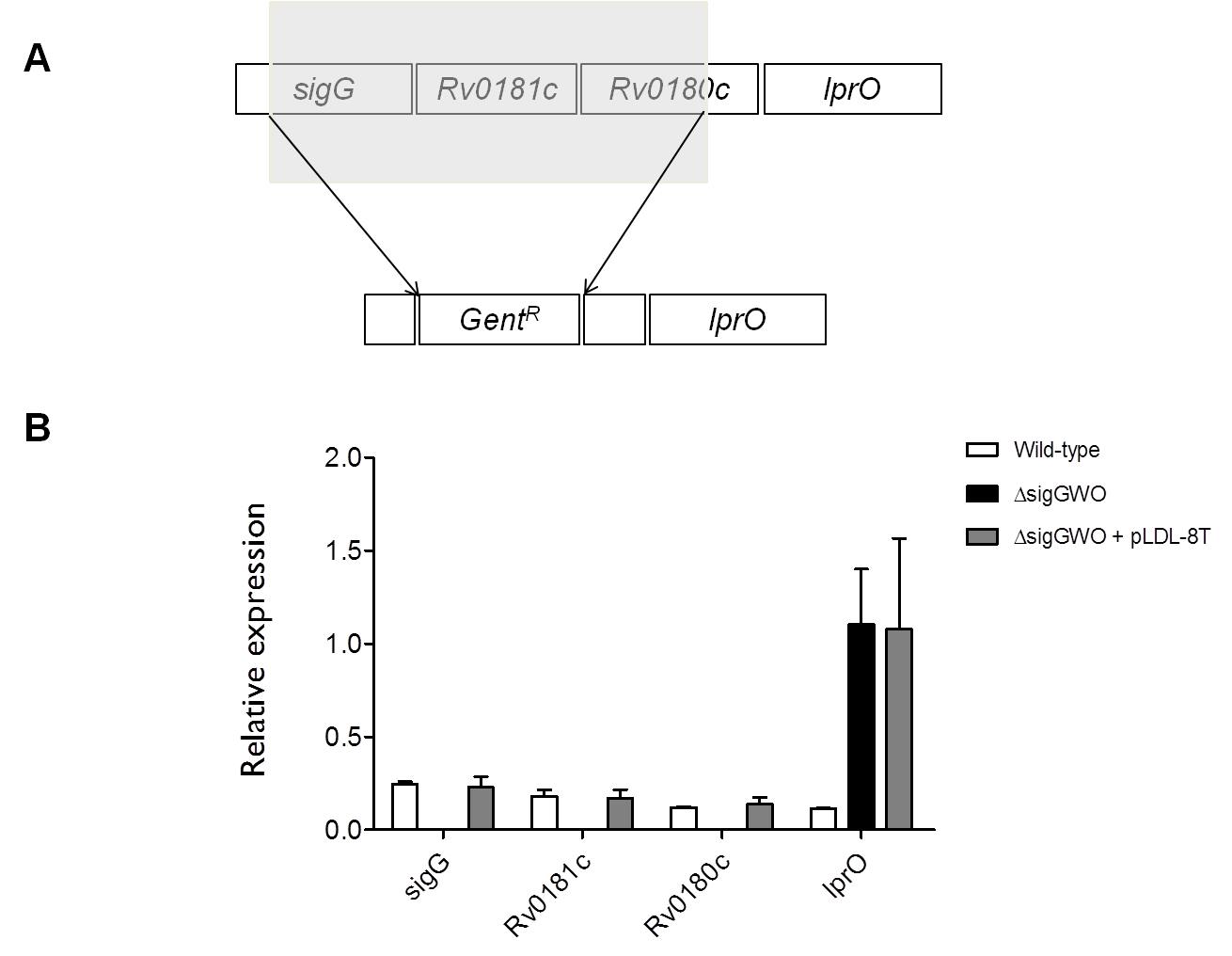
**

**Supplementary Figure 1. qRT-PCR confirming deletion of the *sigG* operon in ∆*sig*GWO.** A. Schematic representation of the region deleted from the *sigG* operon (grey shading) and replaced with a Gentamycin resistance cassette (Gent^R^). B. Expression of *sig*G, *Rv0181c,* *Rv0180c* and *lpr*O analysed by qRT-PCR in wild-type H37Rv, Δ*sigG*WO mutant and Δ*sigG*WO complement (containing pLDL-8T). Data shows expression normalised to *sig*A, mean + standard deviation for 3 biological replicates.

**Supplementary Figure 2.** Quercetinase assay analysing activity of Rv0181c against quercetin. Absorbance of Quercetin at OD_384_ was measured over time in the presence (squares) or absence (circles) of recombinant His-Rv0181c protein. Absorbance decreased more rapidly in the presence of His-Rv0181c indicating that this protein is able to break down quercetin. Data shows absorbance values at different time points calculated as a percentage of the initial absorbance value, the mean and standard deviation of three replicate experiments. Linear regression analysis showed the slopes of the curves to be significantly different, P = 0.02.

**Supplementary Figure 3. ∆*sigG*WO was susceptible to Tween 80 but this was not due to the absence of *sigG*.** Susceptibility of the ∆*sigG*WO mutant (circles) to Tween 80 compared to wild-type *M. tuberculosis* H37Rv (squares) and to two complement strains, where either the whole *sigG* operon (triangles) or only *sigG* (diamonds) were replaced, was determined using an Alamar Blue viability assay. Data represent the mean and standard deviation of three biological replicates.

**SUPPLEMENTARY REFERENCES**

1. **Adams, M., and Z. Jia.** 2005. Structural and biochemical analysis reveal pirins to possess quercetinase activity. J Biol Chem **280:**28675-82.

2. **Lougheed, K. E., D. L. Taylor, S. A. Osborne, J. S. Bryans, and R. S. Buxton.** 2009. New anti-tuberculosis agents amongst known drugs. Tuberculosis **89:**364-70.

3. **Papavinasasundaram, K. G., C. Anderson, P. C. Brooks, N. A. Thomas, F. Movahedzadeh, P. J. Jenner, M. J. Colston, and E. O. Davis.** 2001. Slow induction of RecA by DNA damage in *Mycobacterium tuberculosis*. Microbiol **147:**3271-9.

4. **Rickman, L., C. Scott, D. M. Hunt, T. Hutchinson, M. C. Menendez, R. Whalan, J. Hinds, M. J. Colston, J. Green, and R. S. Buxton.** 2005. A member of the cAMP receptor protein family of transcription regulators in *Mycobacterium tuberculosis* is required for virulence in mice and controls transcription of the *rpfA* gene coding for a resuscitation promoting factor. Mol Microbiol **56:**1274-86.

5. **Springer, B., P. Sander, L. Sedlacek, K. Ellrott, and E. C. Bottger.** 2001. Instability and site-specific excision of integration-proficient mycobacteriophage L5 plasmids: development of stably maintained integrative vectors. Int J Med Microbiol **290:**669-75.

6. **Stover, C. K., V. F. de la Cruz, T. R. Fuerst, J. E. Burlein, L. A. Benson, L. T. Bennett, G. P. Bansal, J. F. Young, M. H. Lee, G. F. Hatfull, and et al.** 1991. New use of BCG for recombinant vaccines. Nature **351:**456-60.
